# “You Think We are in the Stone Age, but We Have Already Made Progress—Where are You?”: A Qualitative Study of Ultra-orthodox Women’s Telemedicine Service Usage in Israel

**DOI:** 10.1007/s10943-024-02212-3

**Published:** 2024-12-29

**Authors:** Irit Chudner, Anat Drach-Zahavy, Batya Madjar, Leah Gelman, Sonia Habib

**Affiliations:** 1 Faculty of Social Welfare and Health Sciences, University of Haifa, Haifa, Israel; 2 Haifa District Health Office, Ministry of Health, Jerusalem, Israel; 3 Tel Aviv, Israel

**Keywords:** Ultra-orthodox, Telemedicine, Primary care, Religious communities, Digital health

## Abstract

This study explores Ultra-Orthodox Jewish women’s attitudes toward video-consultation usage in Israeli primary care settings. In-depth interviews were conducted with twenty-two women from diverse Ultra-Orthodox communities in Israel, using interpretative phenomenological analysis. Despite traditionally limited digital tool usage, participants showed readiness for video-consultations’ adoption through dedicated ‘kosher’ medical devices. Key motivations included after-hours accessibility, convenience, and privacy, while barriers involved cultural stigma and technology concerns. Healthcare organizations should develop dedicated telemedicine devices aligned with religious values, offering insights for implementing culturally sensitive services for religious minority groups.

## Introduction

### Telemedicine and Its Benefits

The implementation of video consultations instead of in-clinic consultations in routine primary care settings has demonstrated substantial benefits: increased accessibility, improved chronic disease management and clinical outcomes, decreased hospitalizations and emergency visits, increased satisfaction with provided services, improved patient waiting times, and reduced costs (Bashshur et al., 2016; Calvo et al., 2014; Chintala, 2023; Izquierdo et al., 2015; Mozes et al., 2022; Reed et al., 2023; Schuttner et al., 2019; Schweiberger et al., 2024; Temesgen et al., 2022; Vosburg & Robinson, 2022). Furthermore, there is an emerging understanding of how different telemedicine modalities can address healthcare disparities among racial and ethnic minority populations, thereby reducing inequities and providing greater access to healthcare (Gibbons & Casale, 2020; Paige et al., 2022; Qureshi et al., 2020; Roghani & Panahi, 2021).

Within Israel’s healthcare system, telemedicine has become increasingly integrated into routine care delivery. Primary care telemedicine is implemented in a hybrid model, combining traditional face-to-face visits with video consultations and other modalities such as store-and-forward correspondence with physicians (Haimi et al., 2020; Hasson et al., 2021; Mozes et al., 2022, 2023; Pilosof et al., 2021). This model of care is delivered by all four Israeli Health Maintenance Organizations and Family Health Centers in communities and is offered at no additional cost to patients. Implementation data indicate successful adoption among the general population, with usage patterns varying across different demographic groups. Current studies indicate that telemedicine adoption in Israel has been particularly strong in urban areas and among younger, technologically proficient populations. However, certain communities, including ethnic and religious minorities, exhibit different patterns of uptake and utilization, highlighting the need for targeted approaches to ensure equitable access to these services.

While the Israeli Ultra-Orthodox community, which comprises 13.6% of the population in Israel (Malach, 2023), could potentially benefit from telemedicine services in general and video consultations in particular, their current usage remains negligible. This disparity stems from different patterns in technology and smart phones usage among the Ultra-Orthodox population compared to general population. While the use of telemedicine in the general population was impacted by the growing usage of smart mobile devices and raised significantly (Israel Internet Association, 2020; Mozes et al., 2022), in the Ultra-Orthodox population has historically prohibited cellular phone use, due to a general suspicion toward technology that characterizes orthodox religious groups (Blondheim & Katz, 2016; Campbell, 2007) and rabbinic prohibition.

### Ultra-Orthodox, Smartphones, and Telemedicine

**Fig. 1:**
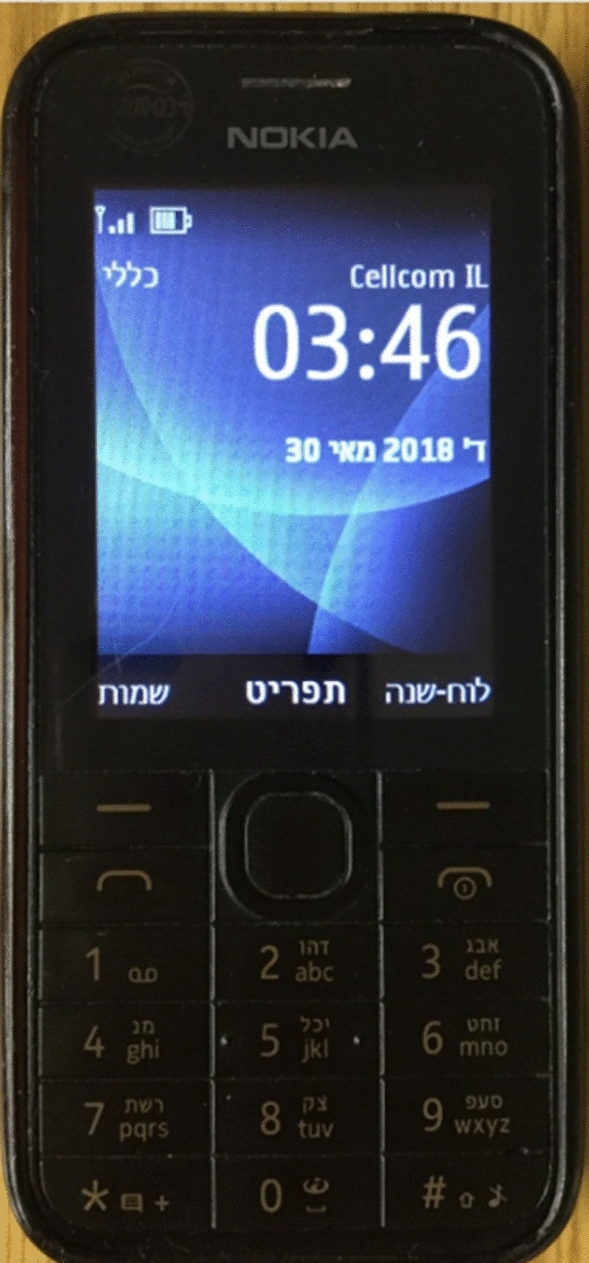
Kosher cell phone

The Ultra-Orthodox or Haredi Jews in Israel constitute a minority group in society that adheres strictly to Halacha rules (Friedman, 1993) and comprises approximately 1,280,000 people. Since the 1950s, this community has perceived itself as a minority at risk of cultural dissolution through assimilation into mainstream Israeli society. Consequently, they have developed various boundary-maintenance mechanisms, including restrictions on technology use, which they perceived as a potential threat to cultural autonomy and religious continuity (Stadler, 2009). Halacha instructions govern most aspects of private and public life for Ultra-Orthodox families, significantly influencing technology usage and health consumption habits. With the proliferation of smartphones, Ultra-Orthodox leaders have intensified efforts to protect the community from what they perceive as threats to the spiritual integrity of community members (Campbell, 2010; Cohen, 2011; Rosenberg & Blondheim, 2021). In response to the infiltration of open internet and smartphones into Ultra-Orthodox society, a group of rabbis, representing several Ultra-Orthodox sub-communities, developed regulatory mechanism to monitor cellular phones use through the Rabbinical Affairs Committee of Communication, an association established in 2005 (Wikipedia, n.d.). This committee prohibited smartphone use and approved only “kosher” phones that lack Internet connectivity. Specifically, a kosher cell phone excludes features commonly found in smartphones such as internet access, web-based applications, camera, radio, music, games, and text messaging capabilities via applications like WhatsApp (Rosenberg & Blondheim, 2021; Rosenberg & Rashi, 2015) [See Fig. 1].

Initially, this ban proved successful, and most devices in Ultra-Orthodox society were either kosher or filtered for content. During this period, as telemedicine programs began emerging in the Israeli public health system, the Ultra-Orthodox community remained unable to access these services due to their use of kosher phones and limited use of internet-enabled computers relative to the general population. However, as stated in the Talmudic law, “Decrees should not be passed if the majority of the people cannot comply with it” (Talmud Bavli Avodah Zara, folio 36a), Ultra-Orthodox people began seeking solutions to reconcile rabbinic prohibitions regarding smartphone use with the demands of everyday life, as smartphones, internet, and computers had become basic platforms for communication and information. Additionally, recent years have seen significantly increased participation of Ultra-Orthodox individuals in the labor market and higher education enrollment (Malach & Cahaner, 2023), while the internet and smartphones have demonstrated value in a wide range of daily applications (Rosenberg & Blondheim, 2021).

**Fig. 2:**
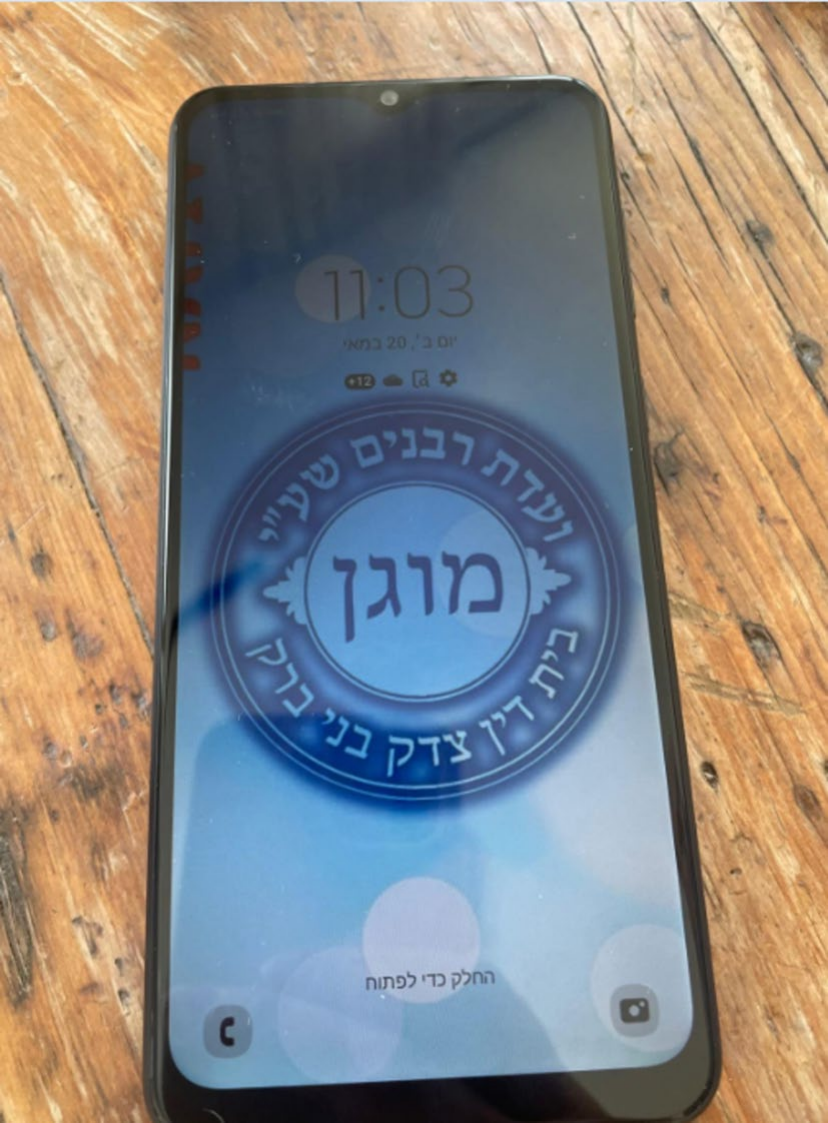
Smart phone with sticked indicating kosher supervision

In recent years, the Rabbinic Committee has approved a modified “kosher smartphone” with content filtering capabilities. This smartphone bears a symbol indicating kosher supervision [See Fig. 2], enabling not only voice communication but also access to camera and limited apps such as GPS, calendar, email, and filtered internet access specifically for work-related needs. This development facilitates a balance between religious observance and modern technological requirements.

Currently, more than 68% of the Ultra-Orthodox population has access to the internet through workplace connections, home networks, and paid internet shops and stations (Malach & Cahaner, 2023). However, smartphone use largely remains unspoken and undisclosed publicly, so there is a lack of formal published statistics regarding smartphone adoption in the Ultra-Orthodox population. Available published data suggest that approximately half of the Ultra-Orthodox public possesses a cell phone (Rosenberg & Blondheim, 2021; Shahar et al., 2023). Despite these technological adaptations, video consultations remain underused, and research examining telemedicine adoption in Ultra-Orthodox society is lacking.

### Ultra-Orthodox Women

Ultra-Orthodox women typically lead busy lives, balancing multiple roles and responsibilities within their families and communities. They commonly marry young and have large families, with an average of 6.6 children per woman (El-Or, 1994; Malach & Cahaner, 2023). These women serve as primary caregivers, managing child-rearing, household duties, and often providing financial support for their families, while their husbands engage in religious studies. This demanding lifestyle creates challenges in accessing health care (Edelstein, 2020; Gershuni et al., 2023) and telemedicine services could particularly benefit these women by providing convenient, time-efficient access to primary healthcare from their homes, enabling video consultation with healthcare providers without the need for traveling or arranging childcare.

While this study focuses on Ultra-Orthodox women in Israel, its implications extend far beyond this specific population. The experiences of Ultra-Orthodox women in accessing video-consultations services offer valuable insights into the needs of other culturally distinct populations worldwide, including religious communities with strict technological guidelines and immigrant communities navigating unfamiliar healthcare systems.

The primary aim of this study is to explore Ultra-Orthodox women’s attitudes and behaviors regarding video-consultation usage in primary care settings, specifically their interactions with family physicians, pediatricians, and nurses in Family Health Stations. Given the global emphasis on telemedicine research in primary care, particularly regarding issues of ethnicity and religion (Gibbons & Casale, 2020; Paige et al., 2022; Qureshi et al., 2020), this study addresses three key research questions. First, how do Ultra-Orthodox women perceive and interact with video-consultation services, particularly considering their traditional limitations on technology use? Second, what cultural, religious, and practical factors facilitate their use of these services? Third, how do communal norms and rabbinic guidance shape their attitudes toward telemedicine, and what implications does this have for service adoption?

Through these investigations, we aim to identify strategies that enhance video-consultations accessibility while respecting religious and cultural values, potentially informing healthcare delivery for both Ultra-Orthodox communities and other cultural minority groups globally.

## Methods

### Research Design

This exploratory study employed an qualitative research design, following current qualitative-methods studies guidelines (Creswell & Poth, 2016) for designing, conducting, and analyzing the data were followed in the study. The study employs a Heideggerian interpretative phenomenological approach (Heidegger, 1962; Lopez & Willis, 2004), chosen for three key considerations. First, it enables understanding of shared attitudes and experiences of a phenomenon among groups of individuals, which is particularly relevant for studying video consultations use in cultural contexts. Second, following Heidegger’s concept of “being-in-the-world” (Dasein), this approach acknowledges that Ultra-Orthodox women’s experiences are inherently shaped by their religious beliefs and community norms. Third, this framework facilitates the development of culturally sensitive practices and policies based on participants’ lived experiences (Creswell & Poth, 2016; Van Manen, 2003). Creswell’s methodological approach was applied within this philosophical framework to systematically analyze and interpret the data. The study received approval from the Institutional Ethics Committee of the University of Haifa. All participants signed the consent form.

### Study Population

Our study population consisted of twenty-two Ultra-Orthodox women with children under 18 years old who had visited their family physician or pediatrician or Family Health Station nurses at least once during the previous year. A purposive snowball non-probabilistic sampling approach was chosen (Creswell & Poth, 2016). Women participating in the study represented diverse Ultra-Orthodox sects, including Lithuanian, Hassidic, and Sephardic groups; various living areas (periphery and center, Ultra-Orthodox and mixed cities); different geographical communities (Jerusalem, Bnei Brak, Zfat, and regions in South and North Israel); and included women who had participated in the *tshuva* movement (Sharabi, 2015), having transitioned from secular life to religious observance. Data saturation was reached after interviewing 19 participants, as no new themes or significant insights emerged in subsequent interviews. Three additional interviews were conducted to ensure complete saturation across different Ultra-Orthodox subgroups, resulting in our final sample size of 22 participants. This sample size aligns with recommended ranges for phenomenological studies (Guest et al., 2006) and proved sufficient to capture the diversity of experiences within the Ultra-Orthodox community.

### Interview Guides

A multidisciplinary team developed the interview guides, comprising a pediatrician, a nurse, two social and behavioral science researchers, and a female Ultra-Orthodox research assistant. The guides were designed to facilitate investigation of attitudes toward video consultations while addressing various aspects of technology and telemedicine usage. Question sampling is presented in Table 1 with full interview guides available in supplementary materials. The first five interviews were transcribed and analyzed to confirm that the protocol was capturing usable data. Additionally, the interview guides explored several topics beyond the scope of this article, mapping attributes and levels of Ultra-Orthodox women’s video-consultation preferences for incorporation into a future Discrete Choice Experiment quantitative study.

**Table 1:** Question sampling

*Community background*
Please tell us about yourself and your community
What aspects of your community are most important to you?
How do these aspects influence your healthcare interactions?
*How could telemedicine services be helpful for your healthcare needs?*
How do you envision incorporating telemedicine into your daily life and community?
What potential benefits and challenges do you see with this technology?
*Technology usage*
How does your community generally view and use computers and telephones?
What types of devices are used at home? (Examples: computers, smartphones, work devices)
*Future vision*
If you could design the perfect telemedicine solution for your community, what features would it include?
What telemedicine services from healthcare providers or insurance companies would best serve your community’s needs?
Please share any other thoughts about telemedicine and healthcare in your community that we haven’t covered

### Procedures

Conducting research within the Ultra-Orthodox community presents unique challenges (Rier et al., 2008), particularly when investigating smartphone ownership and usage, which remains forbidden in some communities and is strictly regulated by rabbinic and societal norms. Consequently, we implemented special methodological considerations: the questionnaire employed formal language, avoiding slang or expressions considered immodest, and an Ultra-Orthodox female research assistant conducted the interviews. The study adhered to guidelines for sensitive topic research (Silverio et al., 2022) including appropriate places for sensitive data collection, indirect questions (Qu & Dumay, 2011), personal diary maintenance and research assistant attending supervision meetings.

Participant recruitment occurred across several cities to ensure geographical and community representation. The research assistant approached women in children’s playgrounds, provided formal letters expressing interest and arranged interview dates and locations. Subsequently**,** interviewees were invited to refer other women from their community who might be interested in participating. All interviews were conducted in Hebrew and lasted 40–120 min. Eighteen out of 22 interviews consented to audio-recording, so their interviews were recorded, enabling verbatim transcription. All data were stored in the researcher’s personal computer with dual password protection.

### Analysis

The research team employed Creswell phenomenological analysis method (Creswell & Poth, 2016), which represents a simplified version of the Steviek-Colaizzi-Keen method (Moustakas, 1994). In alignment with our Heideggerian phenomenological framework, the analysis sought to understand both individual experiences and their broader cultural-religious context. The analytical process proceeded in several stages. First, researchers engaged in reflective discussions regarding their personal attitudes toward Ultra-Orthodox women’s use of telemedicine. This step aimed to set aside the researchers’ preconceptions, ensuring results emerged directly from participants’ responses. Subsequently, researchers independently conducted initial readings of interview transcripts. During the second reading, they performed horizontalization of the data, identifying significant nonrepetitive, non-overlapping statements regarding Ultra-Orthodox women’s perceptions of video consultations.

Significant statements were grouped into larger “meaning units”—themes, accompanied by textual descriptions and supporting quotations. Following independent analysis, researchers convened to compare and discuss their findings ultimately reaching consensus on themes and their textual descriptions. Structural description for each theme was added to enrich the “essence” (El-Or, 1994) of telemedicine experience, encompassing how they used, which types of telemedicine service they accessed, and the context in which video consultations might be utilized.

## Results

The study included twenty-two Ultra-Orthodox women, whose demographic and background characteristics are presented in Table 2.

**Table 2:** Interviewed UO women’ characteristics

Characteristic	N = 22
Gender, Female	22 (100%)
Mean age	32.4
Marital status	Married (100%)
Number of children	4.7
Community	Sephardic (29%), Hasidic (40%), Lithuanian (31%)
Living area	North (24%), Center (40%), Jerusalem area (18%), South (18%)
*Baalot Tshuva* (revival movement)	3 (14%)
Type of education	Religious (65%), Religious and Regular (35%)
Years education achieved	15.7
Working	22 (100%)
Smart phone ownership (Research Assistant assumption based on interview)	*More then* 11 (50%)
Health maintenance organization affiliation*	C (42%), Mac (33%), Meu (24%), Leu (1%)

*C Clalit, *Ma* Maccabi, *Me* Meuhedet, *L* Leumit

Analysis revealed five main themes and seventeen subthemes emerging from the data collected, as presented in Table A3. These themes comprise: (1) Smartphone usage growth; (2) Telemedicine adoption; (3) Usage Motivators; (4) Usage Barriers; (5) Adoption stigma.

The first theme, ‘*Smartphone usage growth’* highlighted increased smartphone adoption in the Ultra-Orthodox community. This theme emerged consistently across interviews, revealing varying levels of openness and implementation of Internet filtering solutions or discreet usage.

**Fig. 3:**
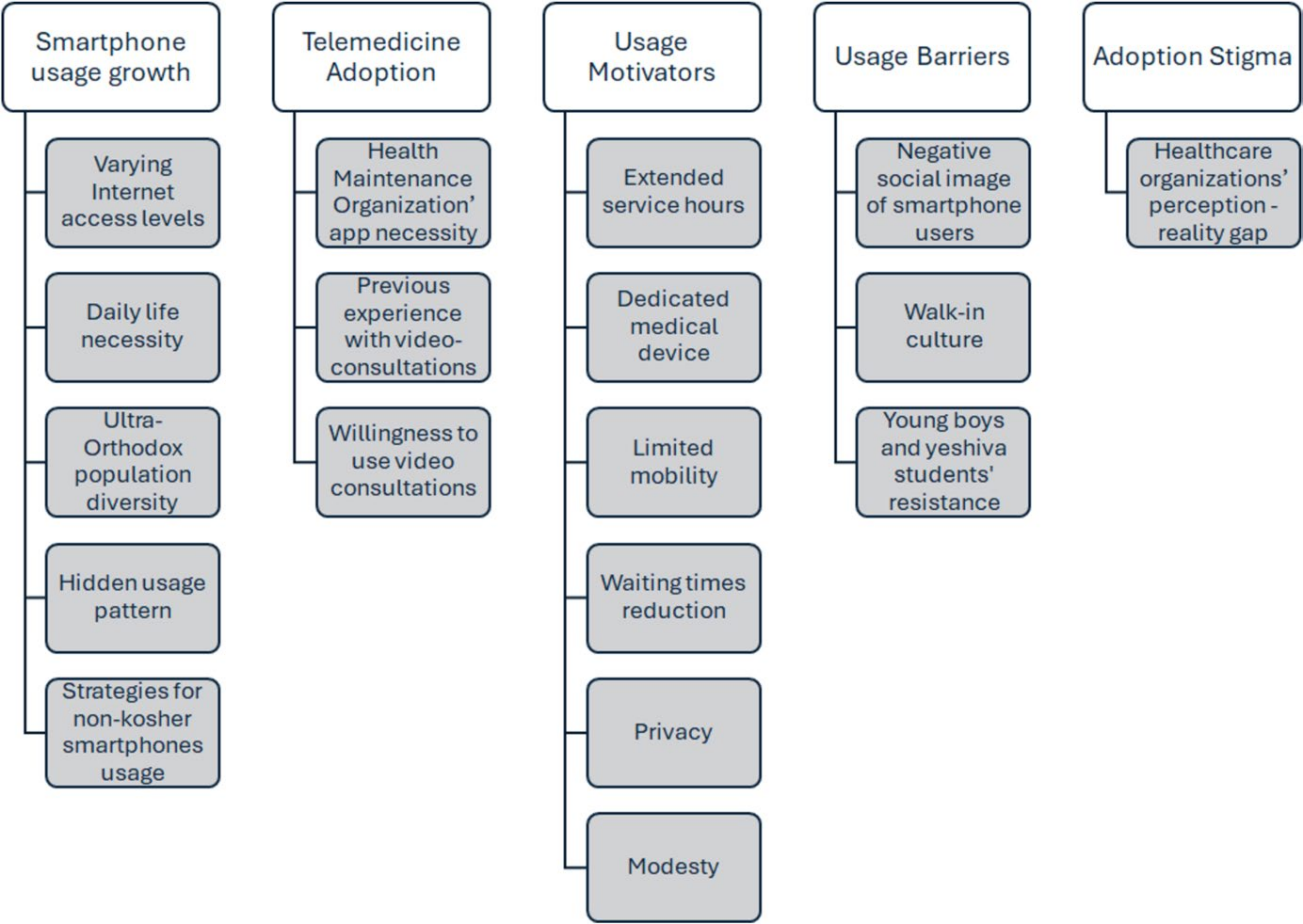
Diagram of themes and sub-themes

Regarding the second theme, ‘*Telemedicine adoption*,’ participants described both their previous and current experiences with telemedicine, primarily through correspondence with family physicians and pediatricians. Notably, five participants reported experience with video consultations specifically for dermatological services and night calling centers. The majority of women, regardless of previous telemedicine experience, expressed interest in using video consultations with primary caregivers, preferring dedicated telemedicine devices and emphasizing the importance of after-hours service availability (Fig. 3).

Analysis of the theme *‘Usage motivators’* identified several key factors: access to medical services outside regular clinic hours (particularly after 20:00), convenience for busy lifestyles, and solutions for limited mobility, as most Ultra-Orthodox women do not drive. Additionally, participants expressed a strong preference for dedicated medical communication devices that are both rabbinically approved and designated exclusively for telemedicine and video-consultation use. The interviewed women emphasized privacy advantages of video consultations, noting that clinic visits risk unwanted encounters with community members and exposure of medical conditions. Additionally, four participants highlighted modesty benefits, particularly the ability to consult with male caregivers remotely rather than in person.

Identified ‘*Usage barriers*’ included cultural norms and negative perceptions of smartphone users, even with rabbinic approval. The barriers also included concerns about exposing children to technology, particularly boys attending religious yeshiva schools, as it was perceived as threatening their ‘spiritual security. Additionally, there is resistance from young boys and yeshiva students against smartphones’ usage for any purpose, as they view it as a threat to their family’s spiritual well-being.

Women frequently discussed the *‘Adoption stigma*’ perpetuated by state health organizations, which they perceived as viewing the Ultra-Orthodox audience as “undeveloped” and ‘outdated’ while making insufficient effort to develop suitable technologies. This revealed a significant gap between health organizations’ perceptions of Ultra-Orthodox readiness for technological adoption and the community’s actual willingness to use these services, suggesting the need for better understanding and tailored approaches. The community has developed various strategies for managing internet and smartphone app usage, including multilevel filtering systems, obtaining special rabbinic approval for work-related smartphone use, and maintaining separate devices for Ultra-Orthodox spaces versus personal and work use.

## Discussion

This research demonstrates a complex relationship between Ultra-Orthodox women and technology, particularly regarding smartphone usage for telemedicine. While smartphone adoption has increased, it varies significantly among subgroups and typically involves varying levels of internet content filtering and strategies to balance rabbinical guidance with practical needs, consistent with previous studies (Rosenberg & Blondheim, 2021; Shahar et al., 2023).

The study identified both shared and distinct motivations for telemedicine use between Ultra-Orthodox women and the general population. Although convenience and time-saving are universal drivers, several factors hold unique significance within this community context. For instance, privacy concerns extend beyond typical healthcare considerations, intersecting with religious and social norms regarding modesty and public disclosure of medical issues.

A substantial proportion of participants indicated that telemedicine technology has become necessary for daily life, particularly for health management through smartphone applications. The analysis revealed both universal and population-specific adoption drivers. While convenience, reduced waiting times, and improved access to care represent universal benefits, Ultra-Orthodox women emphasized unique considerations: video consultation with male physicians (addressing modesty concerns), minimal exposure in public waiting rooms (preserving privacy within tight-knit communities), and healthcare management after fulfilling work and family obligations (reflecting their distinct dual-role challenges). Moreover, the preference for dedicated devices visibly distinguishable from regular smartphones exemplifies specific cultural needs for demonstrating community technology standards adherence.

These population-specific findings present strategic opportunities for healthcare organizations. The development of targeted telemedicine services that incorporate both universal and culturally specific needs could enhance engagement with traditionally underserved communities while respecting religious and cultural values. Specifically, dedicated telemedicine devices could serve as a blueprint for engaging other technology-conscious religious communities.

Evidence indicates growing acceptance of telemedicine, demonstrated through increased correspondence with caregivers via smartphone apps and Health Maintenance Organization internet sites, particularly regarding after-hours video consultations. Participants predominantly expressed willingness to conduct video consultations through special devices exclusively dedicated to caregiver communication, characterized by closed systems preventing open internet exposure and distinct appearances from regular smartphones. This finding underscores the significance of electronic device visibility in Ultra-Orthodox communities and its influence on usage patterns (Rosenberg & Blondheim, 2021). Our findings regarding willingness to use telemedicine appear to diverge from Nariah Ben-Shahar’s (2007) study of Ultra-Orthodox women’s internet perceptions, where participants indicated that ‘only a few things cannot be done without the Internet.’ This apparent contradiction may be attributed to our specific focus on health management and telemedicine, which participants viewed as essential rather than discretionary internet use.

As the first study of its kind examining Ultra-Orthodox women’s attitudes and behaviors toward telemedicine use, this research contributes valuable insights for religion and health technologies researchers and practitioners across health organizations, with implications extending beyond Israel to other societies and health systems. While centered on Ultra-Orthodox women, the findings offer broader insights into implementing telemedicine services among various culturally distinct and communal populations worldwide.

The Ultra-Orthodox population represents one of several ethnic and religious minorities that have experienced limited access to telemedicine services in recent years, including older populations within minority religious groups, indigenous populations, conservative Muslim communities, immigrant communities, and the Amish in the United States. A comparative analysis of Ultra-Orthodox and Amish communities proves particularly enlightening in understanding how traditional religious communities navigate technological change while preserving their values. Both groups have implemented similar strategies: utilizing modified devices (kosher phones versus plain phones), establishing designated technology spaces (internet cafes versus community phones), and maintaining clear boundaries between acceptable and unacceptable uses. Notably, Ultra-Orthodox women demonstrate greater flexibility in adopting health-related technologies, potentially due to their urban setting and workforce integration. These adaptations illustrate how religious minorities can selectively incorporate modern technologies while preserving cultural distinctiveness (Neriya-Ben Shahar, 2017). The development of ‘kosher’ versions of modern technology offers a potential blueprint for other traditional communities seeking to balance technological advancement with cultural preservation.

## Limitations

This study has several limitations. While qualitative methodology was appropriate for its exploratory nature, and the sample encompassed women from all three Ultra-Orthodox groups with characteristics representative of the broader UO population, the sample size remains limited. Additionally, our snowball sampling approach, though effective for accessing this hard-to-reach population, potentially resulted in an overrepresentation of technology-accepting participants. To address this limitation, we diversified our initial recruitment across geographical areas and Ultra-Orthodox subgroups, specifically targeting more conservative communities. Future studies could employ multiple recruitment methods, including partnerships with religious institutions and healthcare providers, to access a broader community spectrum. Furthermore, quantitative research would be necessary to generalize findings to the entire Ultra-Orthodox population. Additional investigation of actual willingness and use patterns would benefit from a study where Ultra-Orthodox women can access rabbinically approved, dedicated telemedicine devices.

## Conclusions

The findings demonstrate perceived technological readiness among Ultra-Orthodox women and their willingness to adopt video-consultation services, highlighting the need for tailored healthcare technology implementation approaches. The development of specialized devices for caregiver communication appears to be a crucial factor in increasing video-consultation adoption among Ultra-Orthodox women. Therefore, policymakers should prioritize developing hardware with distinctive appearances and software that restricts access to unacceptable internet content.

## Appendix

See Table

**Table 3:** Women quotations contributing to themes

#	Theme	Sub-Theme	Quotations
1	Smartphone usage growth	Varying Internet access levels	‘It’s not what it used to be, young people and especially those who work have smartphones.’ *(P15)* ‘There are many filtering solutions nowadays. I have a phone with WAZE with the highest level of filtering. It’s kosher.’ *(P11)* ‘I know there’s a WhatsApp group for young mothers in the neighborhood.’ *(P2)* ‘There are populations that are very strict in their faith but also open and they have phones with different filtering solution available today.’ (*(P12)*
		Daily life necessity	‘Let’s clarify. The rabbis guide us and we ourselves don’t want to use a non-kosher phone. However, […] today it’s difficult to completely avoid using phones and people can’t abide by this decree.’ *(P22)* An interviewee who has filtered internet at home, as well as on her smart device apps for Health Maintenance Organization, banks, waze, Moovit (buses app): ‘It’s technically difficult for me to manage without it. It saves a lot of actions, availability and time instead of physically going somewhere, instead of calling.’ *(P12)*
		Ultra-Orthodox population diversity	‘It’s very different between Lithuanians and Hasidim. For example, among Lithuanians there are working women who drive and also have phones, among Hasidim for example it’s not acceptable at all for a woman to drive and there’s a different attitude if she walks around with a phone in places.’ *(P3)* ‘The problems and prohibitions are different from community to community, and also what’s related to using all kinds of computers for medical purposes.’ *(P6)*
		Hidden usage pattern	‘It is not acceptable like that, for example, like you put the phone on the table to put the phone on the table in our community’ *(P7)* ‘We are not talking about having a smart phone even if it is approved. This is between my husband and me using the phone’ *(P1)* ‘The children do not need to know that there is a phone without filtering at all at home’ *(P9)* ‘Use [smart phone] where it’s needed and in other places don’t take out the device. Don’t take it out in synagogues and weddings.’ *(P13)* We don’t take out a device [smart phone] when it is unnecessary because there’s stigma. We also don’t want to encourage other people that it’s okay to walk around with such a device, it’s still a Hasidic community.*’ (P1)*
		Strategies for non-kosher smartphone usage	‘I keep two phones, one kosher and one regular filtered.’ *(P11)* ‘When the principal of my son’s yeshiva asked why I have a non-kosher number [there is possibility to distinguish between kosher and non-kosher phones by their number] I said it’s because I work in a mixed [religious and secular] workplace and I have rabbinic approval for it.’ *(P7)* ‘I use regular smart phone but it’s filtered and has special sticker on it so it is kosher and people can see the sticker, you can see’ (see Image 2)’ *(P4)*
2	Telemedicine adoption	Health Maintenance Organization app necessity	I have filtered approved smart phone. It is kosher. It has only few necessary things: waze, pango (parking app), bank’ app and HMO’ app.’ *(P12)* I use [HMO app]. It makes appointments, search for doctor services, requests for prescriptions…’ *(P20)*
		Previous experience with video consultations	‘I used an online doctor clinic at 12 at night because the other services weren’t working at that hour.’ *(P12)* I’ve already used a video call with a dermatologist for both myself and my child. I submitted a request through the app for a video appointment and it was scheduled within about 48 h. I connected once from home on mobile with the child together. I showed the camera on the leg, the child didn’t need to speak.’ *(P13)* ‘I used it. You can upload a photo for consultation about skin rashes’*(P5)* ‘I understand that this video call is a medical matter, that it’s for medical purposes—it’s not that I opened the screen to watch a movie…’ *(P21)*
		Willingness to use video consultations	‘It’s very important [video-consultations, it will make things much easier for our families. Instead of going to make an appointment, waiting in the clinic. The inconvenience that you leave the children alone at home or take them with you.’ *(P8)* ‘It’s a good service, from my perspective there’s no problem using video with physician and the child through mobile.’ *(P12)* ‘I would use video with doctor, if it is for medical purposes only.’ *(P16)*
3	Usage motivators	Extended service hours	‘This is a population that doesn’t have much time. I work my first shift at work, then the second shift at home, and after children are going to bed, I start to take care of things, as for example mine and my husband health related tasks’ *(P11)* ‘If you think Tel Aviv is a non-stop city then no, Bnei Brak is a real non-stop city you can buy shoes there at 11 at night, we are very busy and need solutions (medical services) after the two shifts of Haredi mothers end, in the evening.’ *(P10)* We [UO women live a busy lifestyle combining work and home, long workday, if I could make a call to doctor with this video after 20:00 it would be good, to sit in some room and take care of health issues’ *(P17)*
		Dedicated medical device	‘If there were some devices that has different appearance from a smart phone, so I would like to use it for calls with doctors’ *(P12)* ‘To make software or an additional electronic device that allows this transmission [video = consultation]. Not something defined through mobile and not through the computer, purely for this. I also think it would be more acceptable, something dedicated and confined to something specific’ *(P19)* ‘A special device proactively distributed to us by Health Maintenance Organization to every insured patient or household that’s with a service that allows video calls easily. A Haredi family would bring such a thing into the home, of course without any option with something else. It would get rabbinical stamp for such a thing.’ *(P17)* A dedicated device purely for the doctor, purely for calls and tests that are needed.’ *(P21)*
		Limited mobility	‘It’s not easy on public transport with children. Not all clinics are within walking distance.’ *(P14)* ‘Haredi women don’t drive, so it is harder for us to get to the clinic’ *(P2)*
		Waiting times reduction	[by using video consultation, I] ‘will not wait half an hour at the clinic’ *(P16)* [if not using video consultation but face-to-face visit] I need to leave the children alone at home—it’s a story of half an hour—an hour at least. By the time you arrive, get up, get off, this whole story takes an hour…. and therefore, for an Ultra-Orthodox woman it is something that makes it easier if she has the option’ *(P18)*
		Privacy	‘Privacy is more important than many other things, and video consultations can help protect it.’ *(P5)* ‘Women sometimes have health issues they feel embarrassed about and may avoid going to clinics for fear of encountering people they know. This is particularly true for Ultra-Orthodox individuals, who tend to be very conservative regarding in-person medical appointments.’* (P9)* ‘In some situations, it may be easier to use video to maintain privacy, rather than risking being seen at a clinic.’ *(P21)*
		Modesty	‘Video will make things much easier for all, even for the husband who doesn’t want to go out with his wife to the clinic, and you know, there, it is mixed with men and women’ *(P2)*
	Barriers to use	Negative social image of smartphone users	‘Even if rabbis approve you can’t ignore for example the sign hanging at the entrance to Betar Illit. You enter and it’s written: Phones are a danger.’ ‘Even if the phone is useful for contacting a doctor and it’s allowed to use it, there isn’t much honor in it. It’s like you’re walking outside with a garbage bag: you have to do it… take out the trash, but there’s no honor in that walk.’ *(P7)*
		Walk-in culture	‘I have no problem with appointments if needed, then in the morning, my husband goes to the pediatrician for consultation (due to physical proximity to where we live), and then there in the educational frameworks We don’t make an appointment, just come to the doctor and wait and they receive me when I need.’ *(P16)* ‘I don’t have to make an appointment, if it’s close to me, I prefer to arrive physically.’ *(P13)* ‘The branch [of the health insurance] is very close to my workplace, so if they don’t answer and I need a close appointment—I just come.’ *(P19)*
		Young boys and yeshiva students’ resistance	‘I can’t fool my children that it’s only for work purposes, they see that I’m on it a lot and not just for work purposes and apparently they have an opinion about it and about me. It’s like a dam absorbing and it’s a struggle.’ *(P11)* ‘Children in yeshivas are the most ‘spiritually protected’ and won’t like that mom uses the smart phone with doctors.’ *(P14)* ‘It can catch on… there will definitely be parents who won’t want to expose children to the screen. There are communities where even if the parent has a device for work purposes, they still teach children in institutions that it’s for dad’s work purposes, and the child isn’t exposed to the device, doesn’t hold it, doesn’t take pictures with it.’ *(P13)*
5	Adoption stigma	Healthcare organizations perceptions-reality gap	“You think we’re in the Stone Age and we’ve already progressed – where are you?” *(P3)* ‘The first thing that can be done for this population is to recognize their desire, the desire of at least some of them to progress technologically and not to look at them as some stigma.’ *(P7)* ‘Communication in my Family station is face-to-face and by phone because the station doesn’t allow video calls. If there was, I would use it.’ *(P19)*

3.
